# The Amplatzer duct occluder (ADOII) and Piccolo devices for patent ductus arteriosus closure: a large single institution series

**DOI:** 10.3389/fcvm.2023.1158227

**Published:** 2023-05-04

**Authors:** Elchanan Bruckheimer, Kristoffer Steiner, Yuval Barak-Corren, Leonel Slanovic, Michael Levinzon, Alexander Lowenthal, Gabriel Amir, Tamir Dagan, Einat Birk

**Affiliations:** ^1^ Section of Pediatric Cardiology, Schneider Children’s Medical, Center of Israel, Petach Tikva, Israel; ^2^ Department of Women's and Children's Health, Karolinska Institutet, Stockholm, Sweden; ^3^ Section of Pediatric Anesthesiology, Schneider Children’s, Medical Center of Israel, Petach Tikva, Israel

**Keywords:** PDA closure, interventional pediatric cardiology, Piccolo, ADOII, AVPII

## Abstract

**Purpose:**

Evaluate Piccolo and ADOII devices for transcatheter patent ductus arteriosus (PDA) closure. Piccolo has smaller retention discs reducing risk of flow disturbance but residual leak and embolization risk may increase.

**Methods:**

Retrospective review of all patients undergoing PDA closure with an Amplatzer device between January 2008 and April 2022 in our institution. Data from the procedure and 6 months follow-up were collected.

**Results:**

762 patients, median age 2.6 years (range 0–46.7) years and median weight 13 kg (range 3.5–92) were referred for PDA closure. Overall, 758 (99.5%) had successful implantation: 296 (38.8%) with ADOII, 418 (54.8%) with Piccolo, and 44 (5.8%) with AVPII. The ADOII patients were smaller than the Piccolo patients (15.8 vs. 20.5 kg, *p* < 0.001) and with larger PDA diameters (2.3 vs. 1.9 mm, *p* < 0.001). Mean device diameter was similar for both groups. Closure rate at follow-up was similar for all devices ADOII 295/296 (99.6%), Piccolo 417/418 (99.7%), and AVPII 44/44 (100%). Four intraprocedural embolizations occurred during the study time period: two ADOII and two Piccolo. Following retrieval the PDA was closed with an AVPII in two cases, ADOI in one case and with surgery in the fourth case. Mild stenosis of the left pulmonary artery (LPA) occurred in three patients with ADOII devices (1%) and one patient with Piccolo device (0.2%). Severe LPA stenosis occurred in one patient with ADOII (0.3%) and one with AVPII device (2.2%).

**Conclusions:**

ADOII and Piccolo are safe and effective for PDA closure with a tendency to less LPA stenosis with Piccolo. There were no cases of aortic coarctation related to a PDA device in this study.

## Introduction

Transcatheter closure of a PDA is a common and straight-forward procedure in the paediatric age group with a variety of devices and methods available. Choice of device is usually determined by a combination of patient age and weight, the morphology of the ductus and the physician's personal preference. Coils were popular due to the ability to use low-profile systems via arterial access in small patients but were often complicated by incomplete closure or embolization (1). First generation occluders usually required larger delivery systems and a venous approach and the presence of a disc or an umbrella which could protrude to the aortic side limiting the use in smaller patients (2–4). The newer generations of the Amplatzer duct occluders, ADOII and Piccolo (previously called ADOII additional sizes), require a 4 or 5 French delivery system and can be implanted from the aortic or venous side (5, 6). Although available for clinical use since 2011, the Piccolo received FDA approval for PDA closure in children with a body weight over 700 grams in 2019 and has proved to be a highly useful device for transcatheter PDA closure of extremely preterm born children (7–11).

The major difference between the ADOII and the Piccolo is the smaller size of the retention discs in the latter which although advantageous in avoiding flow disturbances may make it less occlusive and prone to embolisation. We report on a large single institution, retrospective study on the outcome of transcatheter PDA closure with the Piccolo in a non-premature cohort.

## Methods

### Patient population

Consecutive patients diagnosed with a PDA who underwent an attempt at transcatheter closure between January 2008 and April 2022 at our institution were identified from the cardiac catheterization database. Patient data, procedural characteristics, hemodynamic and angiographic findings, echocardiographic findings, and clinical status were recorded from the patient records. All aortic angiograms were reviewed by the same physician (KS). Data was collected retrospectively. The study was approved by the Institutional Review Board. Informed, written consent was obtained before each procedure.

### Devices

The ADOII and Piccolo (St Jude Medical, St. Paul, Minnesota) devices have been described previously [reference]. The ADOII Is available in diameters of 3, 4, 5 and 6 mm and lengths of 4 and 6 mm with retention disks 6 mm larger than the device waist. Piccolo is similar but with smaller retention disks which are 4, 5.25 or 6.5 mm when device diameters are 3, 4 or 5 mm, respectively. The Piccolo is also available in a length of 2 mm. The symmetrical design offers the possibility to deploy the Piccolo devices through a 4Fr. delivery sheath either from the aortic or the pulmonary side and the disks, altered from curved to flat, in addition to their occlusive properties, anchor the device on both sides while minimizing risk of protrusion.

### Catheterization

Procedures were performed typically under general anesthesia due to patient age. Following percutaneous access, biplane aortography was performed in right anterior oblique (RAO) and lateral planes and the PDA was closed using standard techniques (12) using a 4–5Fr delivery system from the aortic or pulmonary side. If the size or shape of the PDA was not appropriate for either of these devices an AVPII plug was used. Device selection was at the sole discretion of the interventionalist. PDA morphology on biplane aortogram was classified using the Krichenko classification and modification suggested by Philip (13, 14). A device was chosen so that the diameter of the waist was approximately twice the size of the narrowest part of the ductus, usually at the pulmonary end. The length of the Piccolo was chosen to suit the ductal anatomy, so that in a conical-tubular shaped ductus the aortic disk would lie deep in the ductus at the tip of the cone and in a tubular ductus at the ampulla without extending in to the aortic lumen. Stability of the device was assessed by a mild push-pull the delivery cable. Aortography was performed after implantation to confirm position and residual leak after device release. Color-Doppler echocardiography was performed the following day before discharge.

### Echocardiography

A complete color-Doppler echocardiogram was performed in all patients prior to catheterization, 1 day after PDA occlusion and on all follow up visits. Pulmonary artery stenosis was defined as echocardiographic evidence of turbulent flow and a Doppler velocity >2 m/s that was not demonstrated on echocardiogram before intervention and when greater than 2.5 m/s the stenosis was defined as significant, similar to the national study from UK and Ireland that used 2 m/s as definition for pulmonary artery stenosis (5). Coarctation of the aorta was defined as a pullback peak gradient above 10 mmHg post device deployment if it was not present before device placement. Presence of a residual shunt was defined as echocardiographic evidence of residual flow across the PDA the day following implantation.

Statistical analysis was performed using Kruskal-Wallis nonparametric Anova test and Dunńs Multiple Comparison Test.

## Results

**Figure 1:**
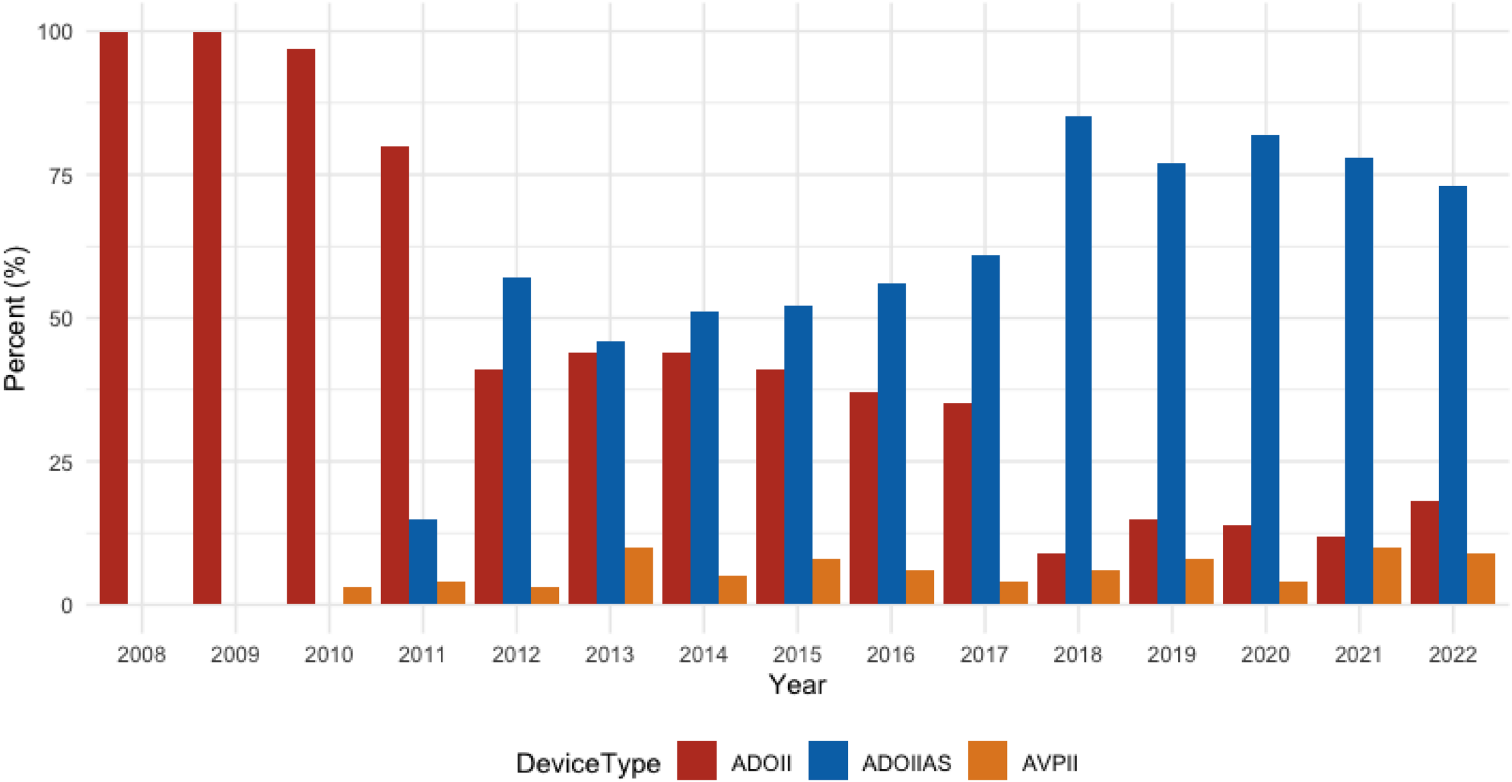
Device use over time 2008–2022. Over this time period, the ADOIIAS became our preferred device, used today in about 80% of all PDA closures.

Between January 2008 and April 2022, 762 patients were referred for PDA closure. Patients and PDA characteristics are summarized in Table 1. The use of Piccolo increased from 10% in 2011 to around 80% of all device-occluded PDA cases in 2021–2022 (Figure 1).

**Table 1:** Patient, PDA and device characteristics. The ADOII patients were smaller than the Piccolo *p* < 0.001 with larger PDA diameters *p* < 0.001.

	ADOII (*N* = 298)	Piccolo (*N* = 420)	AVPII (*N* = 44)	Overall (*N* = 762)
Sex				
Female	206 (69.1%)	270 (64.3%)	33 (75.0%)	509 (66.8%)
Male	92 (30.9%)	150 (35.7%)	11 (25.0%)	253 (33.2%)
Age (years)				
Mean (SD)	3.46 (4.07)	5.03 (4.53)	3.09 (7.74)	4.31 (4.66)
Median [Min, Max]	1.90 [0, 31.8]	3.50 [0, 24.9]	1.00 [0, 46.8]	2.60 [0, 46.8]
Missing	1 (0.3%)	0 (0%)	1 (2.3%)	2 (0.3%)
Weight (kg)				
Mean (SD)	15.8 (13.7)	20.5 (15.8)	11.4 (12.5)	18.1 (15.1)
Median [Min, Max]	11.4 [1.80, 84.0]	14.5 [1.20, 92.0]	8.00 [3.50, 67.0]	13.0 [1.20, 92.0]
Min. diameter (mm)				
Mean (SD)	2.32 (0.479)	1.89 (0.378)	3.07 (0.663)	2.12 (0.538)
Median [Min, Max]	2.25 [1.30, 4.20]	1.80 [1.20, 3.50]	2.90 [2.00, 5.30]	2.00 [1.20, 5.30]
Length (mm)				
Mean (SD)	6.16 (1.43)	7.03 (2.42)	9.08 (2.98)	6.81 (2.24)
Median [Min, Max]	6.00 [3.20, 11.2]	6.50 [2.00, 15.2]	8.60 [4.50, 18.5]	6.30 [2.00, 18.5]
PDA type (Krichenko)				
a	273 (91.6%)	238 (56.7%)	13 (29.5%)	524 (68.8%)
b	2 (0.7%)	0 (0%)	0 (0%)	2 (0.3%)
c	19 (6.4%)	158 (37.6%)	16 (36.4%)	193 (25.3%)
d	3 (1.0%)	7 (1.7%)	0 (0%)	10 (1.3%)
e	1 (0.3%)	13 (3.1%)	1 (2.3%)	15 (2.0%)
f	0 (0%)	4 (1.0%)	14 (31.8%)	18 (2.4%)
Complications				
Embolized	2 (0.7%)	2 (0.4%)	0 (0%)	4 (0.5%)
LPA stenosis	4 (1.3%)	1 (0.2%)	1 (2.3%)	6 (0.8%)
None	292 (98.0%)	418 (99.5%)	42 (95.5%)	752 (98.7%)

The patients who underwent closure with ADOII were significantly younger and smaller than the Piccolo patients. The minimal PDA diameter in the ADOII group was significantly larger than that of the Piccolo and since the mean device diameter was similar for both groups the device:PDA ratio was significantly larger for the Piccolo device group. There were significantly more Krichenko type-C PDAs in the Piccolo group compared to ADOII.

### Complications and follow up

The closure rate at follow up was similar for all devices at 99.6%–100%. All patients were discharged the following day with normal distal pulses palpated and with no venous or arterial complications.

#### Piccolo

There were four implantation failures, two in a type A PDA and two in a type C. In these cases, the Piccolo was demonstrated to be unstable *before release*. The device was retrieved without releasing in all cases and the PDA was closed successfully with an AVPII 6 mm device. There were two device embolizations, both to the right pulmonary artery, which were successfully retrieved, and the PDA was closed with an AVPII 6 mm with no further complications. On mean follow up 8.4 ± 15.2 months, one patient had mild left pulmonary artery stenosis on echocardiography with a maximal velocity of 2.5 m/s. There were no cases of aortic flow disturbance or significant LPA stenosis.

#### ADOII

There were two cases of device embolization (ADOII 4-4, ADOII 5-4) to the right pulmonary artery, one patient was treated with surgical PDA closure the same day. In the other patient the device was successfully retrieved and the PDA was closed using an ADOI 8-6 device. On mean follow up 11.9 ± 20.4 months, there were four cases of LPA stenosis, three mild and one significant stenosis which underwent balloon dilation 4 years later with a good result. There were no cases of aortic flow disturbance. One patient suffered from endocarditis and a residual shunt after PDA closure with an ADOII 5-4 mm device. The shunt eventually disappeared on echocardiographic follow up 14 months after implantation. Summary of all complications by weight of patient is found in Table 2.

**Table 2:** All major complications by weight in kg.

Weight (kg)	Total count	Embolized	LPA stenosis
0–4	5	1 (20.0%)	0 (0%)
5–10	244	2 (0.8%)	2 (0.8%)
11–15	233	1 (0.4%)	1 (0.4%)
16–20	105	0 (0%)	1 (1.0%)
21–25	50	0 (0%)	0 (0%)
25–30	30	0 (0%)	1 (3.3%)
31–40	25	0 (0%)	0 (0%)
>40	70	0 (0%)	1 (1.4%)

#### AVPII

AVPII was used for PDA closure in 44 patients who were smaller and younger than the Piccolo and ADOII patients. On a mean follow up of 7.4 ± 13.4 months there was one complication. A 7-month-old patient (6 kg), who underwent PDA closure with an 8 mm AVPII device, had significant LPA stenosis and underwent balloon angioplasty of the left pulmonary artery with a 6 mm Savvy balloon (SAVVY® Cordis, Miami Lakes, United States). On follow up, the patient demonstrated a perfusion distribution of 30% to the left lung and 70% to the right lung.

## Discussion and limitations

This retrospective study demonstrates that the Amplatzer ductal occluders are safe and effective devices for the transcatheter closure of a patent ductus arteriosus. The lower profile Piccolo device was used in small and larger patients with smaller PDA diameters with an excellent closure rate and very few complications. The major concerns of embolization and residual leak due to its smaller retention disks were not supported when a device:PDA diameter of approximately 2:1 was maintained. In the one case of embolization a review of the angiogram demonstrated the narrowing of the PDA to be dynamic in the RAO plane with a diameter of 3.5 mm which was overlooked at the time of choice of device. A relatively high rate of PDA device embolizations of 2.6% has been reported in a meta- analysis (15). This was not our experience in our cohort (*n* = 4, 0.5%). The reasons for this may relate to our routine use of biplane angiography which enhances the identification and sizing of the narrowest ductal diameter. Since the PDA is often not necessarily viewed best in the lateral plane and can be foreshortened, the 40 degree RAO plane is a good angle for allowing a supplemental view of the PDA morphology. Using both angled views simultaneously can demonstrate fixed or dynamic narrowest ductal diameters more easily.

**Figure 2:**
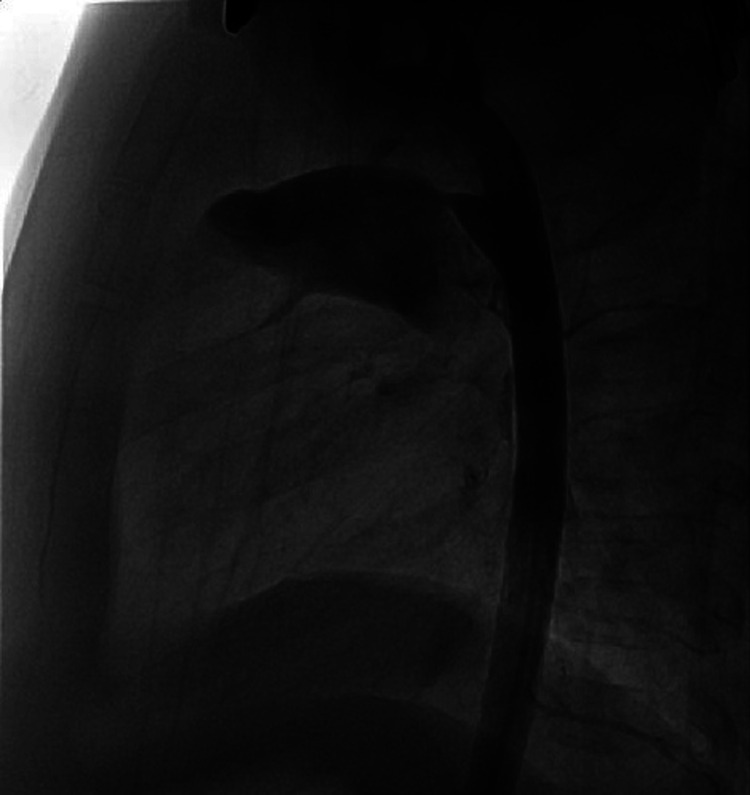
Conical PDA before closure, lateral aortogram.

**Figure 3:**
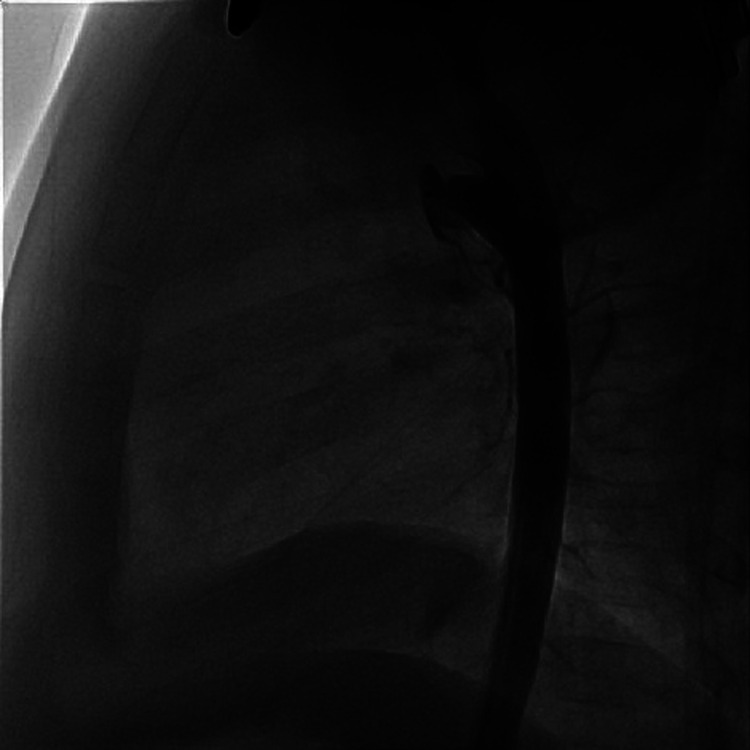
Conical PDA after closure, lateral aortogram.

Once the diameter is established, when using the Piccolo device the delivery is from the aortic side as we have described in our previous report. In this approach the pulmonary disk is extruded completely in the main pulmonary artery (MPA) and approximated to the end of the delivery sheath so that the central body of the device is restricted in the sheath. When pulling the sheath back no further part of the device is released until the pulmonary disk becomes concave, indicating that it is up against the pulmonary arterial wall. While maintaining tension, withdrawal of the sheath releases the body of the Piccolo device in the ductus and aortic ampulla without any part of the body in the narrowing. If the body of the device is in the narrowed part of the ductus it can “stent” the ductus open and possibly make embolization more likely. In a conical ductus [Krichenko A] we use the largest diameter and shortest length Piccolo device so that it fills up the peak of the cone with the device's aortic disk deep in the ampulla providing both excellent closure, even in a moderate ductus, with no peri-device leak and preventing embolization (Figures 2, 3). This approach also prevents any significant pulmonary or aortic flow disturbance. When the ductal diameter is greater than 3 mm we generally used an AVPII device since this is a more robust device with larger diameters available with smaller disks than the ADOII.

When using the ADOII we rarely used the 6 mm long device for PDA closure since the disks are more likely to stand proud of the aortic or pulmonary wall which could cause flow disturbance and inferior closure. In type C ductal anatomy, elongation deformity of the aortic disk causes it to have less traction on the wall and it can prolapse into the ductus and embolize. In this type of ductal anatomy, we recommend the use of a Piccolo or an AVPII.

LPA stenosis of some degree has been reported after transcatheter PDA closure especially in patients below 4 kg body weight and a larger PDA minimal diameter (16). However, in premature and small infants the stenosis is usually transient since the Piccolo device is implanted within the ductus. In larger and older patients the pulmonary disk is placed in the pulmonary lumen and often, in the case of the ADOII, the edge of the disk lies partially across the take-off of the LPA. While a very mild degree of stenosis/flow disturbance may be unavoidable, in our series a significant stenosis was quite unusual, occurring in only six cases (6/762, 0.8%, 4 mild and 2 severe stenoses). This is probably related to the extensive use of the Piccolo device and a higher procedural age. Many authors report on the obvious advantages of this device in extremely premature infants (7–10), however, we and others (17–21) believe that the Piccolo is also very useful for closing small and moderate ductus in all age groups and has become our first choice for transcatheter PDA closure of these ducts. This observation can be seen in the bar chart documenting our choice of device selection over the study period (Figure 1).

The limitations of this report are it being retrospective and a non-randomized comparison of devices, however the relatively large numbers of consecutive patients referred for transcatheter PDA occlusion lends support to our observations.

## Conclusions

ADOII and Piccolo are safe and effective for PDA closure with a tendency to less LPA stenosis with ADOIIAS devices.

### Impact on daily practice

Transcatheter closure of a PDA is a common procedure that should result in effective closure and minimal complications. In our opinion, the Piccolo device is a safe and effective device for transcatheter PDA closure and is our preferred choice for transcatheter closure of small to moderate PDAs in all age groups. This technique has replaced coil closure of PDA in our institution. We prefer a retrograde approach through a 4Fr system with device waist-to-PDA diameter ratio of greater than 2:1 with a length that places the aortic disc inside the diverticulum.

## Data Availability

The raw data supporting the conclusions of this article will be made available by the authors, without undue reservation.
